# Primary care Providers’ approaches to cervical cancer screening in Muslim females

**DOI:** 10.1016/j.pmedr.2023.102126

**Published:** 2023-02-04

**Authors:** Sondos Al Sad, Radhika Pandit, Nooralhuda Alhashim, Mahmoud Abdel-Rasoul

**Affiliations:** a Women Health Primary Care Center, Family and Community Medicine Department, University of California San Francisco, 2356 Sutter St, San Francisco, CA 94115, USA; b The Ohio State University College of Medicine, 370 W 9th Ave, Columbus, OH 43210, USA; c Northeast Ohio Medical University, 4209 St, OH-44, Rootstown, OH 44272, USA; d The Ohio State University College of Medicine, Center for Biostatistics, 1800 Cannon Drive Columbus, OH 43210, USA

**Keywords:** Health equity, Preventive screening, Muslim patients, Public health

## Abstract

The utilization of the Papanicolaou (Pap) test and the human papillomavirus (HPV) vaccine has significantly decreased rates of cervical cancer and related mortality. Disparities in receiving these preventive screenings are scarcely studied in Muslim females. Our study explores primary care providers’ (PCP) approaches to cervical cancer screening in Muslim females. We created a cross-sectional Qualtrics survey using convenience sampling of PCPs who perform Pap tests in central Ohio. Recruitment emails were disseminated via departmental email listservs. We had 200 analyzable responses and 78% of respondents reported having Muslim patients. Bivariate analysis was used to identify predictors of providers’ approaches. Providers younger than 35 years obtained a sexual history from Muslim females less frequently, family medicine providers were more likely to obtain a sexual history from Muslim females, and gynecologists were more likely to offer the HPV vaccine to Muslim females. Providers who counseled patients about Pap tests (P<0.001) and HPV modes of transmission (P<0.004) were more likely to offer cervical cancer screening for Muslim females. Our findings suggested that providers’ age and specialty may be predictors of proactive cervical cancer screening and prevention in Muslim females and that there is a gap between current guidelines and preventive clinical practices regarding the HPV vaccine and transmission counseling.

## 1 Introduction

The Pap test for cervical cancer screening has resulted in a significant decrease in cervical cancer-related mortality (Solomon et al., 2007). Since 1980, medical societies and associations recommended initiating cervical cancer screening with the onset of sexual activity, regardless of the patient’s age. In 2012, revised guidelines were issued to address the harms caused by false-positive findings and subsequent invasive management modalities (Smith et al., 2013). The American College of Obstetrics and Gynecology (ACOG), U.S Preventive Services Task Force (USPSTF), and American Cancer Society (ACS) recommended that screening should not be initiated prior to age 21 and extended the time interval between cervical cytology screenings for low-risk women to every 3 years. Cytology and co-testing for human papillomavirus (HPV) are now recommended every 5 years for women over 30 years of age (American College of Obstetricians and Gynecologists (ACOG), 2012). Despite this, some providers continue to perform annual cervical Pap tests (Saraiya et al., 2010).

Religious factors that impact patients’ healthcare decisions and providers’ perspectives and approaches to these factors are understudied (Padela and Curlin, 2013). Current guidelines do not explicitly address special populations including females who have never been sexually active or Muslim women who only engage in religiously compliant sexual interactions. A literature review on cervical cancer incidence in patients who have not had vaginal intercourse yielded only case reports implying the rarity of its occurrence (Pestana et al., 2015). As of 2018, for every 100,000 women, 8 new cervical cancer cases were reported and 2 women died of this cancer in the US (U.S. Cancer Statistics Working Group, 2021). Although there are no current estimates of cervical cancer rates for Muslim women in the US, cervical cancer mortality is reported for Muslim countries with high numbers of immigrants to the US, notably Pakistan, Bangladesh, Iran, Iraq, and Somalia. Cervical cancer mortality rates for recent female immigrants likely mirror their home countries (Duttagupta et al., 2004, Pew Research Center, 2011).

A growing number of studies are exploring the difficulties of performing Pap tests on Muslim women (Mina Matin et al., 2004). These studies often highlight modesty and lack of reproductive health discussions as obstacles (Johnson et al., 2008, Yosef, 2008, Vu et al., 2016), which healthcare providers must navigate to adequately provide preventive screenings and therapies (Odeh Yosef, 2008), Healthcare providers often prioritize efficiency in their clinical encounters and may overlook patient preferences in exchange for expedient care. Muslim women attribute their reluctance to participate in cervical cancer screening to their perception that medical providers are not sensitive to their values, specifically the importance of modesty (Lipson et al., 1995). Cervical cancer screening for preventive purposes is perceived as unnecessary due to limited health literacy or lack of active symptoms (Salman, 2012). Moreover, low-risk Muslim women and girls may be more likely to attend their annual well-woman visits if they understand that undergoing a pelvic examination is not mandated (Domar, 1986). Muslim women do not expect their providers to be acquainted with all aspects of their beliefs, but they do appreciate providers who demonstrate empathetic inquisition and provide culturally aware care (Simpson and Carter, 2008).

Education about the role of HPV in cervical cancer incidence and the impact of HPV vaccines on primary prevention is another counseling tool for cervical cancer screening encounters (Drolet et al., 2019). HPV is an oncogenic virus that is primarily transmitted via sexual contact. The association between certain high-risk HPV genotypes and the development of cervical cancer is well-established and has shaped the most recent ACS screening guidelines (Lees et al., 2016). Host susceptibility and psychosocial factors such as persistent HPV infection, multiple sexual partners, older age at exposure, smoking, unprotected sex, and poor health literacy propel HPV’s neoplastic potential and progression to cervical cancer and other pathologies (Burd, 2003). Although the HPV vaccine is proven to be prophylactic and revolutionary in preventing cervical cancer, its utilization varies nationwide, and offering the vaccine to patients up to 45 years of age is limited in healthcare settings (Luna et al., 2013, Castellsagué et al., 2011). Patient awareness of the HPV vaccine’s benefits is dependent upon provider knowledge and approach to patient education. Counseling under-resourced patients about HPV morbidity and vaccine information may be challenging (Wong, 2009, Berkowitz et al., 2015) due to the lack of healthcare access and resources in low-income regions (Wong, 2009). Nevertheless, it serves as a less invasive method for cervical cancer prevention than a pelvic examination or Pap test. HPV vaccine counseling serves as primary cancer prevention and can be used as a proxy or an entry to cervical cancer screening discussions for all patients, especially those who may be guarded towards receiving a pelvic examination.

Muslim women fall at the intersection of many social determinants of health and often have their religious preferences for modesty and provider’s gender concordance overlooked and excluded from shared decision-making. Our study aims to explore providers’ approaches to cervical cancer screening and prevention in Muslim females.

## 2 Materials and methods

We created a 29-item online self-administered survey. The study met The Ohio State University Institutional Review Board’s guidelines for the protection of human subjects regarding safety and privacy and was documented as an exempt study. We used convenience sampling of clinicians who perform Pap tests including physicians, nurse practitioners, and physician assistants in family medicine (FM), adolescent medicine/pediatrics, obstetrics and gynecology(OB/GYN), and internal medicine (IM). Survey dissemination was managed using Qualtrics, which has a built-in feature that prevents duplicate entries by detecting cookies from the same device. We recruited study participants by utilizing social media platforms (Twitter, Facebook, Instagram) and medical department listservs in central Ohio that provided a summary of the study and instructions for anonymously completing the online survey. Survey distribution occurred between Feb 1st - June 1st, 2021. There is an unknown denominator since the reach of social media and email listservs is not measurable; while our response rate seems comparable to survey-based studies, it was not feasible to calculate it.

### 2.1 Survey instrument

Our survey items were created de-novo to explore providers’ approaches to cervical cancer screening in Muslim females. The 29 survey items included demographics (Q1-5) and clinical background information (Q6-9) as well as questions regarding provider clinical approach (Q10-17, Q26-29), beliefs (Q18-22), and encountered patient preferences (Q23-25) (See Appendix A).

We used Chi-square or Fisher’s exact tests when relevant for subgroup comparisons of survey responses. All hypothesis testing was two-sided and conducted at 5 % type 1 error rate (alpha = 0.05). Survey responses were collected using Qualtrics (Qualtrics, Provo, UT, 2020). Statistical analyses were conducted using SAS version 9.4 (SAS Institute, Cary, NC).

## 3 Results

### 3.1 Participants’ demographics

We had 200 responses to analyze by June 1st, 2021. Providers who answered the survey were mostly older than 35 years old (51.5%), female (71%), White (64%), married (60.5%), and had <10 years of clinical experience (60%). Respondents’ specialties were OB/GYN (32.5%), FM (30.5%), IM (17.5%), and Adolescent medicine/Pediatrics (8.5%). Less than a quarter of them are employed in large physician groups (23%) (Table 1).

**Table 1:** Participants’ Characteristics (n = 200).

	No.*	% ^$^
All Participants	200	(100.0)
Age (yrs) N = 186		
18–25	11	(5.5)
26–34	88	(44.0)
35–44	44	(22.0)
45–54	17	(8.5)
55–64	20	(10.0)
≥65	6	(3.0)
Gender (N = 186)		
Female	142	(71.0)
Male	43	(21.5)
Non-binary	1	(0.5)
Race (N = 186)		
White/Caucasian	128	(64.0)
South Asian (Pakistani, Indian, Bengali)	28	(14.0)
Black/African American	11	(5.5)
Arab/MENA (Middle East, North Africa)	8	(4.0)
Asian (Chinese, Japanese, Filipino, Korean, Indonesian)	7	(3.5)
Otherⱡ	4	(2.0)
Ethnicity (N = 186)		
Non-Hispanic/Latino	183	(91.5)
Hispanic/Latino	3	(1.5)
Marital Status (N = 186)		
Married	121	(60.5)
Single	57	(28.5)
Divorced	5	(2.5)
Separated	2	(1.0)
Widowed	1	(0.5)
Clinical degree ^ (N = 178)		
MD/MBBS	131	(65.5)
APRN	25	(12.5)
DO	21	(10.5)
PA	1	(0.5)
Years in clinical practice (N = 178)		
10–14 years	22	(11.0)
15–19 years	7	(3.5)
5–9 years	36	(18.0)
<5 years	84	(42.0)
≥20 years	29	(14.5)
Type of Practice (N = 178)		
Hospital-based	95	(47.5)
Large group (6 or more physicians)	46	(23.0)
Small group (5 or fewer physicians)	12	(6.0)
Private Practice	10	(5.0)
Other	15	(7.5)
Clinical Specialty (N = 178)		
Obstetrics & Gynecology	65	(32.5)
Family practice	61	(30.5)
Internal medicine	35	(17.5)
Med/Peds	12	(6.0)
Adolescent medicine	5	(2.5)
Perform cervical pap tests (N-=166)		
Yes	161	(80.5)
No	5	(2.5)
Muslim patients in practice (N = 166)		
Yes	156	(78.0)
No	2	(1.0)
I am not sure	8	(4.0)
% of female patients identified as Muslims (N = 166)		
<25 %	130	(65.0)
25 %-50 %	9	(4.5)
0	1	(0.5)
I do not know	26	(13.0)

*Missing is not included in numbers.

$ Percentages are calculated from N = 200.

ⱡOther Race included: Caucasian and Native American, Columbian, & West Asian.

^ MD/MBBS (Doctor of Medicine/Bachelor of Medicine, Bachelor of Surgery), APRN (Advanced Practice Registered Nurse), DO (Doctor of Osteopathic Medicine), PA (Physician Assistant).

### 3.2 Descriptive data

Most clinicians (78%) in our study care for Muslim patients, yet Muslim patients make up less than a quarter of their patient population (65%). Only 1 in 5 providers (20%) reported never identifying the religion of their female patients before preparing them for a Pap test. Three of four providers would personally “always” or “often” obtain a sexual history from female patients before preparing them for a Pap test and are less likely to “always” do so in Muslim female patients (74% in non-Muslim female patients vs 65.6% in Muslim female patients) (Table 2). Almost one-third of providers (27%) were neutral to the statement, “Muslim female patients are more likely to refuse a cervical Pap test due to chastity reasons compared to non-Muslim female patients” and 22% disagreed with the statement.

**Table 2:** Providers’ approach to patients (Muslim vs Non-Muslim female patients).

**How often do you personally obtain a sexual history before preparing female patients for a pap smear?**	**Non-Muslim Females (N = 166)** N (%)*^$^	**Muslim Females** **(N = 166)** **N (%)**
Always	97 (48.5 %)	92 (46.0 %)
Often	51 (25.5 %)	39 (19.5 %)
Sometimes	13 (6.5 %)	23 (11.5 %)
Rarely	4 (2.0 %)	10 (5.0 %)
Never	1 (0.5 %)	2 (1.0 %)

**How often do you offer the HPV vaccine (Gardasil) to ____ older than 26 years of age who report no history of vaginal penetration or intercourse?**	**Non-Muslim Females (N = 139)** **N (%)**	**Muslim Females** **(N = 147)** **N (%)**
Missing		
Always	41 (20.5 %)	38 (19.0 %)
Often	32 (16.0 %)	18 (9.0 %)
Sometimes	28 (14.0 %)	23 (11.5 %)
Rarely	21 (10.5 %)	30 (15.0 %)
Never	17 (8.5 %)	4 (2.0 %)
I don’t currently have Muslim female patients	N/A	34 (17.0 %)

*Missing is not included in numbers.

$ Percentages are calculated from N = 200.

**Fig. 1:**
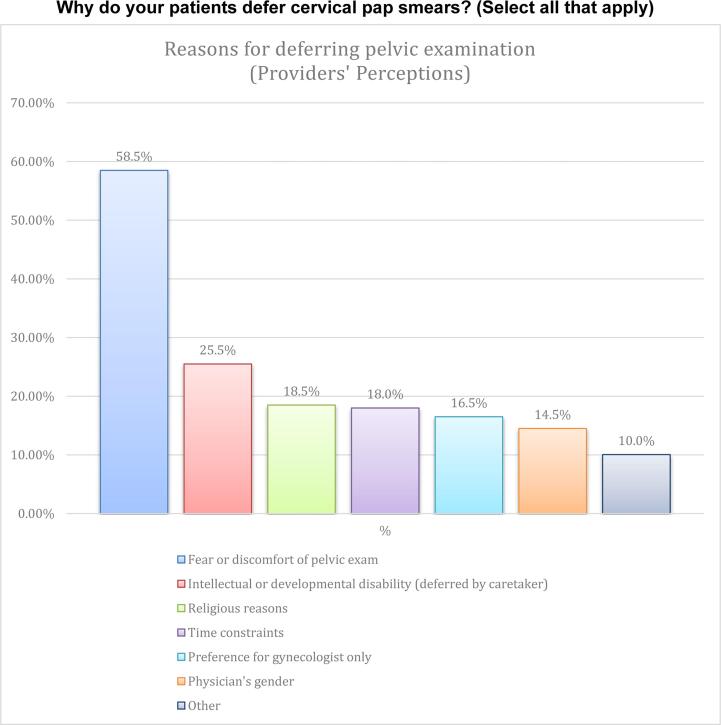
Why do your patients defer cervical pap smears? (Select all that apply).

Most of the providers reported being likely to accommodate gender-concordant care in general (69 %) and a comparable likelihood was seen for religious reasons (68.5 %). Clinicians reported that female patients are more likely to defer pelvic examinations due to fear, disability, religious reasons, preference for OB/GYN, physician gender, and time constraints respectively (Fig. 1).

Most participants (82%) answered “yes” to being familiar with current ACOG and USPSTF cervical cancer screening guidelines and reported following current guidelines either often (20%) or always (61%). Only 1 in 5 providers would “always” offer HPV vaccination to female patients older than 26 years of age with no history of vaginal intercourse or penetration.

Most providers reported believing that cervical cancer is mainly caused by HPV exposure (72.5%); however, only 26.5% of providers disagreed with the statement “I believe cervical HPV acquisition can only occur through vaginal intercourse”. Only 13% of providers report always discussing modes of HPV transmission other than vaginal intercourse, with almost 1 in 10 reporting never counseling patients for various modes of HPV transmission.

### 3.3 Bivariate analysis

Providers younger than 35 years of age are less likely to follow a belief system that values deferring internal pelvic exams due to either perceptions or concerns about the acceptability of this type of exam within the religious tradition (p < 0.009) or the state or practice of refraining from extramarital or from all sexual intercourse (p < 0.003). Providers younger than 35 years of age also reported less frequency of obtaining a sexual history from Muslim patients before the pelvic examination (p < 0.005). Family medicine providers are more likely to obtain a sexual history from Muslim female patients prior to a Pap test compared to other specialists (p < 0.022) (Table 3).

**Table 3:** Bivariate associations between predictor variables and accommodating requests for female providers specifically due to religious tradition.

**Variable**		**Extremely likely (n = 123)**	**Likely (n = 14)**	**Unlikely (n = 3)**	**Extremely Unlikely (n = 3)**	**P value**
**Age**	<35	68 (88.3 %)	6 (7.8 %)	1 (1.3 %)	2 (2.6 %)	0.662
	35+	55 (83.3 %)	8 (12.1 %)	2 (3.0 %)	1 (1.5 %)	
**Race**	White	89 (85.6 %)	9 (8.7 %)	3 (2.9 %)	3 (2.9 %)	0.563
	Other	34 (87.2 %)	5 (12.8 %)	0 (0.0 %)	0 (0.0 %)	
**Clinical Specialty**	Adolescent/Meds-Peds	11 (100.0 %)	0 (0.0 %)	0 (0.0 %)	0 (0.0 %)	0.617
	Family practice	47 (88.7 %)	4 (7.5 %)	1 (1.9 %)	1 (1.9 %)	
	Internal medicine	23 (92.0 %)	1 (4.0 %)	0 (0.0 %)	1 (4.0 %)	
	Obstetrics & Gynecology	42 (77.8 %)	9 (16.7 %)	2 (3.7 %)	1 (1.9 %)	
**Marital Status**	Ever Married	86 (83.5 %)	11 (10.7 %)	3 (2.9 %)	3 (2.9 %)	0.628
	Single	37 (92.5 %)	3 (7.5 %)	0 (0 %)	0 (0 %)	
**“Muslim female patients are likely to refuse pap tests due to chastity”**	Strongly agree	7 (87.5 %)	0 (0.0 %)	0 (0.0 %)	1 (12.5 %)	0.016
	Agree	32 (84.2 %)	5 (13.2 %)	1 (2.6 %)	0 (0.0 %)	
	Neutral	50 (94.3 %)	3 (5.7 %)	0 (0.0 %)	0 (0.0 %)	
	Disagree	31 (83.8 %)	5 (13.5 %)	0 (0.0 %)	1 (2.7 %)	
	Strongly disagree	3 (50.0 %)	1 (16.7 %)	1 (16.7 %)	1 (16.6 %)*	
**“Value deferring pelvic exams due to religious traditions”**	Yes	31 (94.0 %)	1 (3.0 %)	0 (0 %)	1 (3.0 %)	0.026
	No	67 (90.5 %)	5 (6.8 %)	1 (1.4 %)	1 (1.3 %)*	
	Maybe	25 (69.4 %)	8 (22.2 %)	2 (5.6 %)	1 (2.8 %)	
**“Value deferring pelvic exam due to refraining from sexual intercourse”**	Yes	19 (90.5 %)	0 (0 %)	1 (4.7 %)	1 (4.6 %)*	0.198
	No	78 (87.7 %)	9 (10.1 %)	1 (1.1 %)	1 (1.1 %)	
	I am not sure	26 (78.8 %)	5 (15.2 %)	1 (3.0 %)	1 (3.0 %)	

*Variables in the table are abbreviated from the survey. In the survey, we asked “Do you follow a belief system that values”: followed by the variable statement.

Gynecologists are more likely to offer the HPV vaccine to women older than 26 years of age (p < 0.033) and to strongly agree with the statement, “I believe cervical cancer is mainly caused by HPV” compared to other specialists (p < 0.002). This held true for Muslim female patients, but to a lesser extent, where gynecologists are most likely to offer the HPV vaccine to Muslim female patients older than 26 years of age (p < 0.005). Interestingly, providers who are more likely to discuss the indications for a cervical Pap test with female patients who report no history of vaginal penetration or intercourse are more likely to offer the HPV vaccine to female patients in general (p < 0.006) and Muslim female patients (p < 0.001) older than 26 years of age. Providers who discuss modes of transmission of HPV other than vaginal intercourse were more likely to offer HPV vaccine to Muslim females older than 26 years of age (p < 0.004); however, this approach did not statistically predict HPV vaccine offering to older non-Muslim female adults.

## 4 Discussion

Our study showed a high rate of compliance and knowledge of the current cervical cancer screening guidelines, yet providers fail to always obtain sexual histories from patients more than half of the time. Furthermore, providers listed fear as the top reason that patients refuse a pelvic examination, listing religious reasons and physician gender as the third and fifth reasons respectively. It will be of benefit to mirror the providers’ perception to that of patients using the same list of reasons in further research.

Of significance, providers’ age, specialty, and the extent of counseling appeared to predict most of their approaches towards cervical cancer screening and prevention in all female patients, particularly Muslims. Provider's specialty is presented in a plethora of studies as a predictor of patient compliance, immunization rate (Koepke et al., 2001, Vadaparampil et al., 2011), implementation of guidelines (Han et al., 2011), and counseling for preventive screening (Gulitz et al., 1998). The impact of provider age scarcely mirrored our findings in the literature (Liebermann et al., 2021) which may attest to the length of clinical expertise and exposure to practice-based learning. The depth and extent of counseling are evident in the literature across many health behaviors; patients are more likely to comply and follow medical advice if they are deliberately informed (DePue et al., 2008, Wee et al., 2005).

In a systematic review, provider-oriented interventions were found to increase cervical cancer screening recommendations by evaluating the provider’s delivery and tailoring screening interventions to the community’s needs rather than a generalizable message (Community Preventive Services Task Force, 2012). Religiosity’s impact on patient and provider perspectives is underrepresented in healthcare disparities (Arousell and Carlbom, 2016). In studies conducted by the Initiative on Islam and Medicine, predictors of screening mammography for breast cancer amongst Muslim women were religious coping mechanisms, perceptions of religious discrimination, age, and years of residence in the US (Padela et al., 2015). Predictors of cervical cancer screening were religious coping mechanisms amongst women as well, those with negative coping mechanisms (thinking that illness is a punishment of God) had lower odds to get Pap tests (Padela et al., 2014). Community-based and provider-oriented interventions may bridge some of the gaps in cancer screening and improve population health.

### 4.1 Pelvic examination

Reflecting on these findings, we believe that building a rapport by personally inquiring about sexual and reproductive health history and transparently counseling patients about the benefits and potential harms of pelvic examination are areas of improvement in clinical practice (O'Laughlin et al., 2021, Hilden et al., 2003).

Current guidelines have evolved over time to reduce the frequency of Pap tests and pelvic examinations to avoid overdiagnosis and potentially harmful interventions (Sawaya et al., 2019, Liverani et al., 2020). Also, the notion that pelvic examination is only intended for cervical cancer screening may need to be further investigated among both clinicians and patients (Stormo et al., 2012) and is worth elaboration in the guidelines, especially the components of a preventative wellness visit (Amir et al., 2014). When a patient refuses a preventive screening test offered by a clinician and their refusal is documented in an electronic health record, they may be labeled as a difficult or challenging patient (Steinmetz and Tabenkin, 2001). This can result in the patient receiving aversive or passive-aggressive care (McPhee et al., 1986). It is important to identify the reasons behind a patient’s deferral of an exam deemed necessary to optimize health outcomes and promote preventive screening (Peterson et al., 2016, Redwood-Campbell et al., 2011). For example, Muslim females who prefer gender concordance or those who have not had vaginal intercourse are more likely to share these reasons if providers take the time to learn their needs and preferences. Two-thirds of our participants were likely to accommodate a request for gender concordance in general and for religious reasons.

Even though 80% of participants reported variably identifying patients’ religion, Muslim female patients consistently received a less attentive approach. One-third of our participants were neutral regarding the statement, “Muslim female patients are more likely to refuse a cervical Pap test due to chastity reasons compared to non-Muslim female patients,” which may manifest in not knowing if this is actually a differentiating reason to defer a pelvic examination for that cohort. Further qualitative research among American Muslim women of all ethnicities is warranted to dissect the role of religion versus culture in defining chastity and provide clinicians with more definitive directions. Most of the studies identified in the literature use “Arab Americans” interchangeably with “American Muslims” (Hammoud and Siblani, 2003) even though Arabs make up less than one-fifth of Muslims worldwide. Furthermore, narrowing this narrative to Islam fails to recognize the embodiment of virginity and its value in various other religions and nationalities in the literature (Rahman, 2018, Bentlage and Eich, 2007). The constant changes in law, medicine, art, religion, and traditions seen in history reflect how chastity and its value have been understood, used, and refined over time (Abboud, 2014).

Nonetheless, many studies focus on patient-reported barriers to cervical cancer screening without addressing provider-centered factors. Although these barriers exist and create disparities in health outcomes, clinicians are not immune to bias or challenges, especially regarding sexuality and reproductive health (Gupta et al., 2020, Williams, 2018, Downing et al., 2007). Our findings demonstrated that younger providers were less likely to follow a belief system that values deferring internal pelvic exams due to perceptions or concerns about the acceptability of this type of exam within the religious tradition, or due to the state or practice of refraining from extramarital or from all sexual intercourse.

Also, younger providers obtained a sexual history from Muslim female patients prior to a Pap test less frequently when compared to non-Muslim female patients (Loeb et al., 2011). This may need further qualitative research to explain the disparity, but it is possible that under-valuing patient preferences may limit provider interest or ability to invest in comprehensive sexual history gathering from that cohort. This finding could also result from an implicit bias towards the “physical chastity” concept embraced by Muslims or other like-minded faith groups (Chapman et al., 2013).

There is a plethora of literature regarding the sociocultural barriers Muslim women face in utilizing sexual and reproductive health services, however, the clinical research may be biased toward the said population due to geopolitical affairs. In a content analysis of more than 2000 MEDLINE abstracts about Muslims in clinical literature by Laird et. al, a couple of major themes were noted; deficient and inaccurate representation of Muslim Americans in research and the skewed portrayal of Islamic traditions as unhealthy and discordant with modernized medical services (Laird et al., 2007). Leaving the impact of these biases unexamined may negatively affect providers’ communication and practice in clinical encounters.

Family medicine providers were most likely to gather sexual history prior to a cervical Pap test, which may be explained by the nature of the longitudinal relationships that family medicine providers have with their patients. This may be of prospective benefit by providing cultural information modules to other primary care specialties and building on this strength in family medicine training. As an example, counseling a group of Iranian women based on a health belief model modified their cervical cancer screening and improved performance (Parsa et al., 2017).

### 4.2 HPV and HPV vaccine

While most of our respondents reported compliance and familiarity with current cervical cancer screening guidelines, only a minority reported recommending the HPV vaccine to their adult female patients older than 26 years of age (20.5 % for non-Muslim females, vs 19 % for Muslim females).

We believe that counseling about HPV and the HPV vaccine is another missing recommendation in the current guidelines, as there is a lack of an explicit statement on factoring in virginity and offering HPV vaccines to adult females older than 26 years of age. The role of the HPV vaccine should prove itself a priority in the prevention of cervical cancer over secondary preventive modalities such as cervical Pap tests (Marcus et al., 2021). Given the recent update in the guidelines; it is recommended to delay the starting age for HPV co-testing to 25 years of age instead of 21 years (Marcus et al., 2021). This recent evolution is a great achievement of clinical efforts in offering the HPV vaccine to males and females. Yet, counseling and offering the HPV vaccine are not consistent nor equitable across the nation (Hirth et al., 2016, Karuri et al., 2017).

Our findings suggest a knowledge gap that may be compounded by a mismatched interpretation of the sociocultural characteristics of Muslim patients. For instance, many participants reported believing that cervical cancer is mainly caused by HPV exposure; however, only a quarter of them disagreed with the statement “*I believe cervical HPV acquisition can only occur through vaginal intercourse*”. The statement may be endorsed due to the fact that age at first heterosexual encounter and number of sexual partners are the most predictive factors to acquiring cervical HPV (Franco et al., 1995), however, this is not the reality of current healthcare exchanges regarding cervical cancer incidence and counseling. Today’s sexuality is stretched beyond vaginal intercourse between the opposite sex; sexual toys, female-to-female (Doull et al., 2018), finger-to-cervical, vertical transmission, and fomite transmissions such as ultrasound probes and specula have been reported (Sabeena et al., 2017). In our study, only 13 % of providers report always discussing modes of HPV transmission other than vaginal intercourse, with almost 1 in 10 reports never counseling patients on the various modes of HPV transmission. Suboptimal counseling may be attributed to deficits in clinical knowledge of modes of transmission (Trucchi et al., 2020), the ability to balance time between clinical services and counseling, and providers’ bias toward women’s sexuality overall (Leung et al., 2019). Literature shows that female patients appreciate knowledgeable physicians that tactfully approach HPV counseling and testing (Qaderi et al., 2021), especially females with intersecting marginalized identities such as Muslim women. Educating patients about the modes of transmission and the importance of prevention through safe sex and HPV vaccines can mend some of the medical mistrust marginalized communities feel (Ho et al., 2021).

OB/GYN specialty was significantly linked to a strong agreement with the statement “I believe cervical cancer is mainly caused by HPV” compared to other specialists, another finding that mirrors the literature and indicates another knowledge gap. The gap may be due to the magnitude of exposure to females as a patient cohort, or focused clinical knowledge versus a broad spectrum in primary care settings. In addition, gynecologists were more likely to offer the HPV vaccine to Muslim and non-Muslim females older than 26 years old. This could be due to the availability of the HPV vaccine in gynecology offices, a higher knowledge base of gynecologists, or practice-enforced metrics. Even so, Muslim female patients were offered HPV vaccination at a lower rate by gynecologists when compared with non-Muslim females. Some Muslim patients may be interested in learning if the indicated vaccines are halal, which is not a common knowledge for most providers (Aziz et al., 2021). Interestingly, providers who are more likely to discuss the indications for a Pap test with female patients who report no history of vaginal penetration or intercourse are more likely to offer the HPV vaccine to female patients older than 26 years of age. Those who discuss modes of transmission of HPV other than vaginal intercourse were more likely to offer HPV vaccine to Muslim females older than 26 years of age. We believe that if a provider can comfortably discuss the modes of HPV transmission and indications for Pap tests, they may be more prepared to navigate encounters with a wide spectrum of patients including those who are reserved. Studies in the literature show that the biggest barrier to HPV vaccines in women being treated for abnormal Pap tests is lack of knowledge (Roane et al., 2021).

Our results indicate that providers rarely counsel patients about modes of HPV transmission, even though most believe cervical cancer is primarily caused by HPV exposure and that vaginal intercourse did not get a full consensus to be the only mode of transmission. This subpar health education and preventive counseling can be remedied by purposeful medical education of both current and future physicians on how to address patients’ needs, thus promoting prevention and healthy changes (Williams, 2002, Daniel et al., 2021).

Cervical cancer continues to burden women and HPV vaccination rates are well below the Healthy People objectives with regional and ethnic disparities (Hirth, 2019). Further attention to the role of healthcare providers’ approaches in delivering equitable counseling and preventive services to Muslim females is warranted.

### 4.3 Strengths

We believe our study offers an equitable and transformative perspective on the accountability of healthcare providers towards all patients and highlights clinicians’ approaches to Muslim females as an underrepresented cohort in clinical research and settings.

### 4.4 Limitations

Our study has the same limitations of cross-sectional design and online-based survey recruitment was challenged by the COVID-19 pandemic in healthcare settings.

## 5 Conclusion

Our study intended to explore potential factors that may impact providers’ approach to cervical cancer screening and prevention in Muslim female patients. Our descriptive data brought valuable directions for future research in bridging the gap in caring for Muslim patients and potentially other faith-based cohorts. Furthermore, our analysis suggested that providers’ age and specialty are predictors of proactive counseling with Muslim females, which may require intentional teaching in medical education and residency training. Further exploration of the impact of adherence to clinical guidelines, approaches to counseling underrepresented cohorts, HPV vaccines in Muslim males, and expanding the study to the national sphere to identify regional factors is merited.

## CRediT authorship contribution statement

**Sondos Al Sad:** Conceptualization, data curation, project administration, resources, supervision, validation, and original and revision writing. **Radhika Pandit:** Project administration, Data curation, Writing- original draft, review and editing. **Nooralhuda Alhashim:** Project administration, Data curation, Writing- original draft, review and editing. **Mahmoud Abdel-Rasoul:** Formal analysis, methodology.

## Declaration of Competing Interest

The authors declare that they have no known competing financial interests or personal relationships that could have appeared to influence the work reported in this paper.

## Data Availability

The authors do not have permission to share data.

## References

[b0005] [Unknown]. (2012). Updated recommendations for client- and provider-oriented interventions to increase breast, cervical, and colorectal cancer screening. Am. J. Prevent. Med. 43(1), 92–96. https://doi.org/10.1016/j.amepre.2012.04.008. 10.1016/j.amepre.2012.04.008 22704753

[b0010] Abboud S. (2014).

[b0015] American College of Obstetricians and Gynecologists (ACOG). (2012). ACOG Practice Bulletin no. 109: Cervical cytology screening. Obstetr. Gynecol. 120(5), 1222–1238. doi:http://10.1097/AOG.0b013e318277c92a.

[b0020] Amir Q., Linda L.H., Russell H., Melissa S., Thomas D.D. (2014). Screening pelvic examination in adult women: a clinical practice guideline from the American College of Physicians. Ann. Internal Med..

[b0025] Arousell J., Carlbom A. (2016). Culture and religious beliefs in relation to reproductive health. Best Pract. Res. Clin. Obstetr. Gynaecol..

[b0030] Aziz, N. A., Sulaiman, S. S., bin Roslan, M. A., Aizat, K. M. A., Yusof, K., & Raof, N. A. (2021). THE HALAL STATUS OF COVID-19 VACCINE: REVISITING THE ROLE OF NATIONAL PHARMACEUTICAL REGULATORY AGENCY (NPRA) AND JAKIM. E-BOOK OF EXTENDED ABSTRACT, 169.

[b0035] Bentlage B.J.Ö.R.N., Eich T. (2007). Hymen repair on the Arabic Internet. ISIM Rev..

[b0040] Berkowitz Z., Malone M., Rodriguez J., Saraiya M. (2015). Providers’ beliefs about the effectiveness of the HPV vaccine in preventing cancer and their recommended age groups for vaccination: findings from a provider survey, 2012. Prevent. Med..

[b0045] Burd E.M. (2003). Human papillomavirus and cervical cancer. Clin. Microbiol. Rev..

[b0050] Castellsagué X., Muñoz N., Pitisuttithum P., Ferris D., Monsonego J., Ault K., Luna J., Myers E., Mallary S., Bautista O.M., Bryan J., Vuocolo S., Haupt R.M., Saah A. (2011). End-of-study safety, immunogenicity, and efficacy of quadrivalent HPV (types 6, 11, 16, 18) recombinant vaccine in adult women 24–45 years of age. Br. J. Cancer.

[b0055] Chapman E.N., Kaatz A., Carnes M. (2013). Physicians and implicit bias: how doctors may unwittingly perpetuate health care disparities. J. General Internal Med..

[b0060] Daniel C.L., McLendon L., Green C.L., Anderson K.J., Pierce J.Y., Perkins A., Beasley M. (2021). HPV and HPV vaccination knowledge and attitudes among medical students in Alabama. J. Cancer Educ..

[b0065] DePue J.D., Goldstein M.G., Redding C.A., Velicer W.F., Sun X., Fava J.L., Rakowski W. (2008). Cancer prevention in primary care: predictors of patient counseling across four risk behaviors over 24 months. Prevent. Med..

[b0070] Domar A.D. (1986). Psychological aspects of the pelvic exam: Individual needs and physician involvement. Women Health.

[b0075] Doull M., Wolowic J., Saewyc E., Rosario M., Prescott T., Ybarra M.L. (2018). Why girls choose not to use barriers to prevent sexually transmitted infection during female-to-female sex. J. Adolescent Health.

[b0080] Downing R.A., LaVeist T.A., Bullock H.E. (2007). Intersections of ethnicity and social class in provider advice regarding reproductive health. Am. J. Public Health.

[b0085] Drolet M., Bénard É., Pérez N., Brisson M., Ali H., Boily M.-C., Baldo V., Brassard P., Brotherton J.M.L., Callander D., Checchi M., Chow E.P.F., Cocchio S., Dalianis T., Deeks S.L., Dehlendorff C., Donovan B., Fairley C.K., Flagg E.W., Gargano J.W., Garland S.M., Grün N., Hansen B.T., Harrison C., Herweijer E., Imburgia T.M., Johnson A.M., Kahn J.A., Kavanagh K., Kjaer S.K., Kliewer E.V., Liu B., Machalek D.A., Markowitz L., Mesher D., Munk C., Niccolai L., Nygård M., Ogilvie G., Oliphant J., Pollock K.G., Purriños-Hermida M.J., Smith M.A., Steben M., Söderlund-Strand A., Sonnenberg P., Sparen P., Tanton C., Wheeler C.M., Woestenberg P.J., Yu B.N. (2019). Population-level impact and herd effects following the introduction of human papillomavirus vaccination programmes: updated systematic review and meta-analysis. Lancet.

[b0090] Duttagupta C., Sengupta S., Roy M., Sengupta D., Bhattacharya P., Laikangbam P., Roy S., Ghosh S., Das R. (2004). Are Muslim women less susceptible to oncogenic human papillomavirus infection? A study from rural eastern India. Int. J. Gynecol. Cancer.

[b0095] Franco E.L., Villa L.L., Ruiz A., Costa M.C. (1995). Transmission of cervical human papillomavirus infection by sexual activity: differences between low and high oncogenic risk types. J. Infect. Dis..

[b0100] Gulitz E., Bustillo-Hernandez M., Kent E.B. (1998). A provider survey. Cancer Pract..

[b0105] Gupta N., Kucirka L.M., Semerjian A., Wainger J., Pierorazio P.M., Herati A.S., Bivalacqua T.J. (2020). Comparing provider-led sexual health counseling of male and female patients undergoing radical cystectomy. J. Sexual Med..

[b0110] Hammoud M.M., Siblani M.K. (2003). Care of Arab Americans and American Muslims. Cross-cultural Med..

[b0115] Han P.K., Klabunde C.N., Breen N., Yuan G., Grauman A., Davis W.W., Taplin S.H. (2011). Multiple clinical practice guidelines for breast and cervical cancer screening: perceptions of US primary care physicians. Med. Care.

[b0120] Hilden M., Sidenius K., Langhoff-Roos J., Wijma B., Schei B. (2003). Women’s experiences of the gynecologic examination: factors associated with discomfort. Acta Obstet. Gynecol. Scand..

[b0125] Hirth J. (2019). Disparities in HPV vaccination rates and HPV prevalence in the United States: a review of the literature. Human Vaccines immunotherapeutics.

[b0130] Hirth J.M., Lin Y.L., Kuo Y.F., Berenson A.B. (2016). Effect of number of human papillomavirus vaccine doses on guideline adherent cervical cytology screening among 19–26year old females. Prevent. Med..

[b0135] Ho I.K., Sheldon T.A., Botelho E. (2021). Medical mistrust among women with intersecting marginalized identities: a scoping review. Ethnicity Health.

[b0140] Johnson C.E., Mues K.E., Mayne S.L., Kiblawi A.N. (2008). Cervical cancer screening among immigrants and ethnic minorities: a systematic review using the Health Belief Model. J. Lower Genital Tract Dis..

[b0145] Karuri A.R., Kashyap V.K., Yallapu M.M., Zafar N., Kedia S.K., Jaggi M., Chauhan S.C. (2017). Disparity in rates of HPV infection and cervical cancer in underserved US populations. Front. Bioscience (Scholar edition).

[b0150] Koepke C.P., Vogel C.A., Kohrt A.E. (2001). Provider characteristics and behaviors as predictors of immunization coverage. Am. J. Prevent. Med..

[b0155] Laird L.D., De Marrais J., Barnes L.L. (2007). Portraying Islam and Muslims in MEDLINE: a content analysis. Soc. Sci. Med..

[b0160] Lees B.F., Erickson B.K., Huh W.K. (2016). Cervical cancer screening: evidence behind the guidelines. Am. J. Obstet. Gynecol..

[b0165] Leung S.O.A., Akinwunmi B., Elias K.M., Feldman S. (2019). Educating healthcare providers to increase Human Papillomavirus (HPV) vaccination rates: a Qualitative Systematic Review. Vaccine: X.

[b0170] Liebermann E., Van Devanter N., Frías Gúzman N., Hammer M.J., Ompad D. (2021). Dominican provider attitudes towards HPV testing for cervical cancer screening and current challenges to cervical cancer prevention in the Dominican Republic: a mixed methods study. J. Cancer Educ..

[b0175] Lipson J.G., Hosseini T., Kabir S., Omidian P.A., Edmonston F. (1995). Health issues among Afghan women in California. Health Care Women Int..

[b0180] Liverani C.A., Di Giuseppe J., Giannella L., Delli Carpini G., Ciavattini A., Magi-Galluzzi C. (2020). Cervical cancer screening guidelines in the postvaccination era: review of the literature. J. Oncol..

[b0185] Loeb D.F., Lee R.S., Binswanger I.A., Ellison M.C., Aagaard E.M. (2011). Patient, resident physician, and visit factors associated with documentation of sexual history in the outpatient setting. J. General Internal Med..

[b0190] Luna J., Plata M., Gonzalez M. (2013). Long-term follow-up observation of the safety, immunogenicity, and effectiveness of Gardasil (TM) in adult women. PLoS One.

[b0195] Marcus J.Z., Cason P., Downs L.S., Einstein M.H., Flowers L. (2021). The ASCCP cervical cancer screening task force endorsement and opinion on the American Cancer society updated cervical cancer screening guidelines. J. Lower Genital Tract Disease.

[b0200] McPhee S.J., Richard R.J., Solkowitz S.N. (1986). Performance of cancer screening in a university general internal medicine practice. J. General Internal Med..

[b0205] Mina Matin BA & Samuel LeBaron MD and PhD (2004) Attitudes toward cervical cancer screening among Muslim Women: a pilot study, Women Health 39(3), 63-77, DOI: 10.1300/J013v39n03_05. 10.1300/J013v39n03_05 15256356

[b0210] Odeh Yosef A.R. (2008). Health beliefs, practice, and priorities for health care of Arab Muslims in the United States: implications for nursing care. J. Trans. Nurs..

[b0215] O'Laughlin D.J., Strelow B., Fellows N., Kelsey E., Peters S., Stevens J., Tweedy J. (2021). Addressing anxiety and fear during the female pelvic examination. J. Primary Care Community Health.

[b0220] Padela A.I., Curlin F.A. (2013). Religion and disparities: considering the influences of Islam on the Health of American Muslims. J. Relig. Health.

[b0225] Padela A.I., Peek M., Johnson-Agbakwu C.E., Hosseinian Z., Curlin F. (2014). Associations between religion-related factors and cervical cancer screening among Muslims in greater Chicago. J. Lower Genital Tract Dis..

[b0230] Padela A.I., Murrar S., Adviento B., Liao C., Hosseinian Z., Peek M., Curlin F. (2015). Associations between religion-related factors and breast cancer screening among American Muslims. J. Immigrant Minority Health.

[b0235] Parsa P., Sharifi F., Shobeiri F., Karami M. (2017). Effects of group counseling based on health belief model on cervical cancer screening beliefs and performance of rural women in Kaboudrahang, Iran. Asian Pacific J. Cancer Prevent.: APJCP.

[b0240] Pestana I., Costa A., Gorgal R., Mota R., Portugal R., Paiva V. (2015). Cervical uterine cancer in a virgin young woman – Case report. J. Obstet. Gynaecol..

[b0245] Peterson E.B., Ostroff J.S., DuHamel K.N., D'Agostino T.A., Hernandez M., Canzona M.R., Bylund C.L. (2016). Impact of provider-patient communication on cancer screening adherence: a systematic review. Prevent. Med..

[b0250] Pew Research Center. (2011). e global Muslim population: Projections for 2010–2030. Washington, DC: Author. Retrieved from www.pewforum.org/future-of-the-global-muslim-populationregional-americas.aspx.

[b0255] Qaderi K., Geranmayeh M., Farnam F., Hasani S.S., Mirmolaei S.T. (2021). Understanding HPV-positive women’s needs and experiences in relation to patient-provider communication issues: a qualitative study. BMC Health Services Res..

[b0260] Rahman S. (2018). Female sexual dysfunction among Muslim women: Increasing awareness to improve overall evaluation and treatment. Sex. Med. Rev..

[b0265] Redwood-Campbell L., Fowler N., Laryea S., Howard M., Kaczorowski J. (2011). 'Before you teach me, I cannot know': immigrant women’s barriers and enablers with regard to cervical cancer screening among different ethnolinguistic groups in Canada. Can. J. Public Health = Revue canadienne de sante publique.

[b0270] Roane B., Mahalingam M., Boitano T., Wall J., Straughn M., Huh W. (2021). Attitudes and knowledge of HPV vaccination in women being treated for an abnormal Pap smear. Gynecol. Oncol..

[b0275] Sabeena S., Bhat P., Kamath V., Arunkumar G. (2017). Possible non-sexual modes of transmission of human papilloma virus. J. Obstet. Gynaecol. Res..

[b0280] Salman K.F. (2012). Health beliefs and practices related to cancer screening among Arab Muslim women in an urban community. Health Care Women Int..

[b0285] Saraiya M., Berkowitz Z., Yabroff K.R., Wideroff L., Kobrin S., Benard V. (2010). Cervical cancer screening with both human papillomavirus and Papanicolaou testing vs Papanicolaou testing alone: what screening intervals are physicians recommending?. Arch. Internal Med..

[b0290] Sawaya G.F., Smith-McCune K., Kuppermann M. (2019). Cervical cancer screening: more choices in 2019. JAMA.

[b0295] Simpson J.L., Carter K. (2008). Muslim women’s experiences with health care providers in a rural area of the United States. J. Trans. Nurs..

[b0300] Smith R.A., Brooks D., Cokkinides V., Saslow D., Brawley O.W. (2013). Cancer Screening in the United States, 2013 A Review of Current American Cancer Society Guidelines, Current Issues in Cancer Screening, and New Guidance on Cervical Cancer Screening and Lung Cancer Screening. CA: a Cancer Journal for Clinicians.

[b0305] Solomon D., Breen N., McNeel T. (2007). Cervical cancer screening rates in the United States and the potential impact of implementation of screening guidelines. CA: a cancer journal for clinicians.

[b0310] Steinmetz D., Tabenkin H. (2001). The ‘difficult patient' as perceived by family physicians. Family Pract..

[b0315] Stormo A.R., Cooper C.P., Hawkins N.A., Saraiya M. (2012). Physician characteristics and beliefs associated with use of pelvic examinations in asymptomatic women. Prevent. Med..

[b0320] Trucchi C., Restivo V., Amicizia D., Fortunato F., Manca A., Martinelli D., Montecucco A., Piazza M.F., Prato R., Tisa V., Ansaldi F., Icardi G. (2020). Italian health care workers’ knowledge, attitudes, and practices regarding Human Papillomavirus infection and prevention. Int. J. Environ. Res. Public Health.

[b0325] U.S. Cancer Statistics Working Group. U.S. Cancer Statistics Data Visualizations Tool, based on 2020 submission data (1999-2018): U.S. Department of Health and Human Services, Centers for Disease Control and Prevention and National Cancer Institute; www.cdc.gov/cancer/dataviz, released in June 2021.

[b0330] Vadaparampil S.T., Kahn J.A., Salmon D., Lee J.-H., Quinn G.P., Roetzheim R., Bruder K., Malo T.L., Proveaux T., Zhao X., Halsey N., Giuliano A.R. (2011). Missed clinical opportunities: provider recommendations for HPV vaccination for 11–12 year old girls are limited. Vaccine.

[b0335] Vu M., Azmat A., Radejko T., Padela A.I. (2016). Predictors of delayed healthcare seeking among American Muslim women. J. Women’s Health.

[b0340] Wee C.C., McCarthy E.P., Phillips R.S. (2005). Factors associated with colon cancer screening: the role of patient factors and physician counseling. Prevent. Med..

[b0345] Williams G.C. (2002). 11: improving patients’ health through supporting the autonomy of patients and providers. Handb. Self-determination Res..

[b0350] Williams D.R. (2018). Stress and the mental health of populations of color: advancing our understanding of race-related stressors. J. Health Soc. Behav..

[b0355] Wong L.P. (2009). Physicians’ experiences with HPV vaccine delivery: evidence from developing country with multiethnic populations. Vaccine.

[b0360] Yosef A.R. (2008). Health beliefs, practice, and priorities for health care of Arab Muslims in the United States. J. Trans. Nurs..

