# Supplementary material for: Primary care Providers’ approaches to cervical cancer screening in Muslim females
Source: Prev Med Rep. 2023 Feb 4;32:102126. doi: 10.1016/j.pmedr.2023.102126 (PMC9958399; doi:10.1016/j.pmedr.2023.102126)
Supplement: Supplementary data 1 [file mmc1.pdf]

Default Question Block

The Ohio State University Consent to Participate in Research

Study Title: *Primary Care Providers Approach to Cervical Cancer Screening Among Muslim Females Refusing Pelvic Examination*

Protocol Number: 2020E1135
Researcher: Sondos Al Sad, MD, MPH
Co-Investigators: Nooralhuda Alhashim, Radhika Pandit

This is a consent form for research participation:
It contains important information about this study and what to expect if you decide to participate.

Your participation is voluntary. Please consider the information carefully. Feel free to ask questions before making your decision whether or not to participate.

Purpose:
This study aims to help us understand the approach of primary care providers towards performing cervical pap smears in Muslim women who refuse internal pelvic examination due to the patients' belief that they are not at risk or do not need this screening because they are monogamous or practice abstinence.

Procedures/Tasks:

Participation in this research will **take approximately 5 minutes**. You will be asked to complete an online survey to tell us about your experiences and opinions performing pap smears.

You may leave the study at any time. If you decide to stop participating in the study, there will be no penalty to you, and you will not lose any benefits to which you are otherwise entitled. Your decision will not affect your future relationship with The Ohio State University.

Risks and Benefits:

Your responses will be used to improve our understanding of physicians' attitudes towards performing cervical cancer screenings on Muslim women who report chastity. It is expected that you will experience no greater risks than those encountered in your day-to-day life.

Privacy and Confidentiality:

Efforts will be made to collect your answers in private; as you will be able to utilize an online platform to answer the survey from your own device and at your chosen location and time. Your name, email, or any other identifying information will not be collected as part of your survey completion, and as a result, investigators will not have access to your personal information.

We will work to make sure that no one sees your online responses without approval. But, because we are using the Internet, there is a chance that someone could access your online responses without permission. In some cases, this information could be used to identify you.

Also, there may be circumstances where this information must be released. For example, personal information regarding your participation in this study may be disclosed if required by state law. Also, your records may be reviewed by the following groups (as applicable to the research):
Office for Human Research Protections or other federal, state, or international regulatory agencies;
The Ohio State University Institutional Review Board or Office of Responsible Research Practices;
The sponsor, if any, or agency (including the Food and Drug Administration for FDA-regulated research) supporting the study.

Future Research:

Your de-identified information may be used or shared with other researchers without your additional informed consent.

Participant Rights:

You may refuse to participate in this study without penalty or loss of benefits to which you are otherwise entitled. If you are a student or employee at Ohio State, your decision will not affect your grades or employment status.

If you choose to participate in the study, you may discontinue participation at any time without penalty or loss of benefits. By agreeing to participate, you do not give up any personal legal rights you may have as a participant in this study.

This study has been approved by OSU IRB and determined Exempt

Contacts and Questions:

For questions, concerns, or complaints about the study you may contact Dr. Sondos Al Sad at: Sondos.AlSad@osumc.edu.

For questions about your rights as a participant in this study or to discuss other study-related concerns or complaints with someone who is not part of the research team, you may contact the Office of Responsible Research Practices at 1-800-678-6251 or hsconcerns@osu.edu.

Providing consent

I have read (or someone has read to me) this page and I am aware that I am being asked to participate in a research study. I have had the opportunity to ask questions and have had them answered to my satisfaction. I **voluntarily agree** to participate in this study. I am not giving up any legal rights by agreeing to participate.

To print or save a copy of this page, select the print button on your web browser.

Please click the button below to proceed and participate in this study. If you do not wish to participate, please close out your browser window.

The following terms used in the survey are defined as :

Pap smear: a sample of cells collected from the female cervix using a speculum for cervical cancer screening.

Muslim Females: Female patients who self-identify as Muslims, reporting adherence to Islamic teachings about religiously sanctioned sexual practices

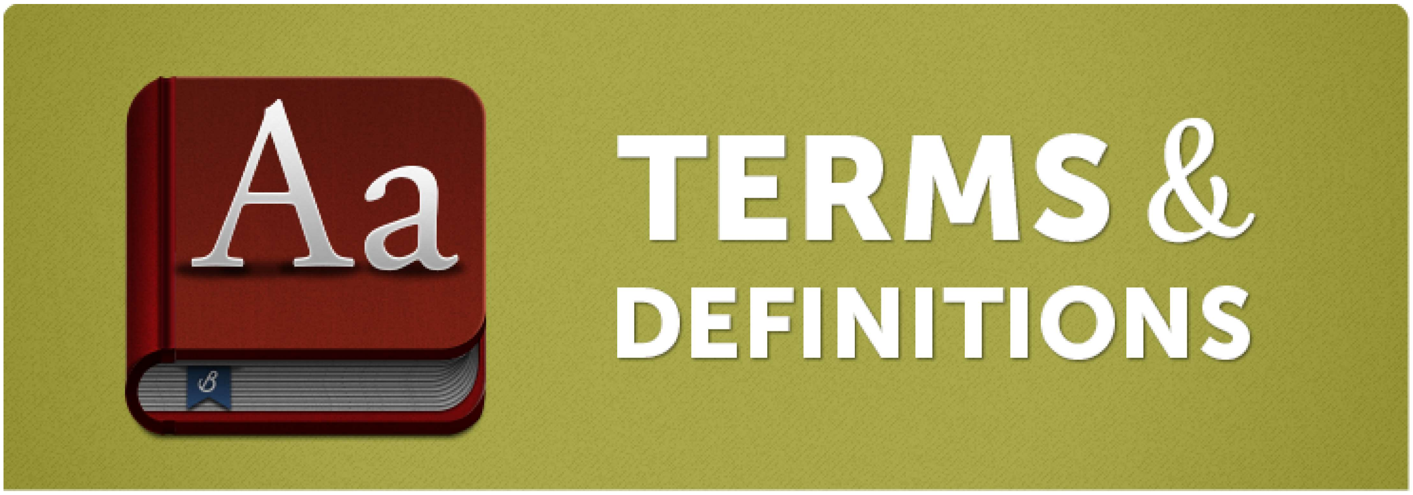

Demographics:

This set of questions aims to help us better understand your demographics as a primary care provider.

What is your age (in years)?

- ○ 18-25
- ○ 26-34
- ○ 35-44

- 45-54
- 55-64
- 65-75
- >=75

What is your gender?

- Male
- Female
- Non-binary

What is your race?

- White/Caucasian
- Black/African American
- South Asian (Pakistani, Indian, Bengali)
- Asian (Chinese, Japanese, Filipino, Korean, Indonesian)
- American Indian/ Alaska Native
- Native Hawaiian/ Other Pacific Islander
- Mixed (more than one race)
- Arab/MENA (Middle East, North Africa)
- __________ Other

What is your ethnicity?

- Hispanic/Latino
- Non Hispanic/Latino

What is your marital status?

- Married
- Single
- Separated
- Widowed
- Divorced

Clinical practice info

The next set of questions aim to help us understand the nature of your clinical practice.

What is your degree?

- MD/MBBS
- APRN
- DO
- PA

How many years have you been in clinical practice?

- < 5 years
- 5-9 years
- 10-14 years
- 15-19 years
- >= 20 years

In which type of practice do you work?

- Private Practice
- Hospital-based
- Small group (5 or fewer physicians)
- Large group (6 or more physicians)

- Other

What is your clinical specialty in primary care?

- Family practice
- Internal medicine
- Obstetrics & Gynecology
- Pediatrics
- Adolescent medicine
- Med/Peds

Providers' Approach

The next set of questions aims to help us understand your knowledge of cervical cancer screening/prevention and performing pap smears in your clinic.

Do you perform pap smears for cervical cancer screening?

- Yes
- No

Are you familiar with the current ACOG and USPSTF cervical cancer screening guidelines for performing pap smears (age, sexual activity, etc.)?

- Yes
- No
- I am not sure

How often do you follow current ACOG and USPSTF guidelines for performing pap smears?

- Always

- Often
- Sometimes
- Rarely
- Never

Do you take care of Muslim patients in your clinic?

- Yes
- No
- I am not sure

Approximately what percentage of your female patients in your practice identify as Muslims?

- 0%
- Less than 25%
- 25%-50%
- 50%-75%
- 75%-100%
- I do not know

How often do you ***personally*** identify the religion of female patients prior to preparing female patients for a pap smear?

- Always
- Often
- Sometimes
- Rarely
- Never

How often do you ***personally*** obtain a sexual history from female patients prior to preparing female patients for a pap smear?

- Always
- Often
- Sometimes
- Rarely
- Never

How often do you ***personally*** obtain a sexual history from **Muslim** female patients prior to preparing female patients for a pap smear?

- Always
- Often
- Sometimes
- Rarely
- Never

Providers' Beliefs

The next set of questions aims to help us understand your attitudes and beliefs towards the impact of refusing pelvic examination due to patients' belief that they are not at risk or do not need this screening because they are monogamous or practice abstinence cervical cancer prevention, screening, and performing pap smears in your clinic.

Please rate the degree to which you agree or disagree with the following statements:

	Strongly agree	Agree	Neutral	Disagree	Strongly disagree
I believe cervical cancer is mainly caused by HPV exposure	○	○	○	○	○

	Strongly agree	Agree	Neutral	Disagree	Strongly disagree
I believe that cervical HPV acquisition can only occur through vaginal intercourse	○	○	○	○	○
Muslim female patients are more likely to refuse a cervical pap smear due to chastity reasons compared to non-Muslim female patients.	○	○	○	○	○

Do you follow a belief system that values deferring internal pelvic exams due to perceptions or concerns about the acceptability of this type of exam within the religious tradition?

- ○ Yes
- ○ Maybe
- ○ No

Do you follow a belief system that values deferring internal pelvic exams due to the state or practice of refraining from extramarital or from all sexual intercourse?

- ○ Yes
- ○ No
- ○ I am not sure

How often do patients defer pap smears out of refusal of internal pelvic exams; due to perceptions or concerns about the acceptability of this type of exam within their religious traditions in your practice?

- Always
- Often
- Sometimes
- Rarely
- Never

How often do patients defer pap smears due to reporting no history of vaginal penetration or intercourse your practice?

- Always
- Often
- Sometimes
- Rarely
- Never

Why do **your** patients defer cervical pap smears? (select all that apply)

- Religious reasons
- Fear or discomfort of pelvic exam
- Intellectual or developmental disability (deferred by caretaker)
- Preference for gynecologist only
- Physician's gender
- Time constraints
- Other
- Not applicable

How often do you discuss the indications for a cervical pap smear with female patients who report no history of vaginal penetration or intercourse?

- Always
- Often
- Sometimes
- Rarely
- Never

How often do you offer the HPV vaccine (Gardasil) to females **older than 26 years** of age who report no history of vaginal penetration or intercourse?

- Always
- Often
- Sometimes
- Rarely
- Never
- Not applicable

How often do you offer the HPV vaccine (Gardasil) to ***Muslim*** females **older than 26 years** of age who report no history of vaginal penetration or intercourse?

- Always
- Often
- Sometimes
- Rarely
- Never
- I don't currently have Muslim female patients

How often do you discuss modes of transmission of HPV other than vaginal intercourse?

- Always
- Often

- Sometimes
- Rarely
- Never

How likely are you to accommodate requests for the gynecologic exam to be performed by a female provider?

- Extremely unlikely
- Unlikely
- Likely
- Extremely likely
- There are no female providers in my clinic

How likely are you to accommodate requests for the gynecologic exam to be performed by a female provider ***specifically due to religious beliefs***?

- Extremely unlikely
- Unlikely
- Likely
- Extremely likely
- There are no female providers in my clinic
